# The Vital Theory of the Causation of Carcinoma

**Published:** 1902-08-30

**Authors:** 


### THE VITAL THEORY OF THE CAUSATION OF CARCINOMA.

In a series of articles which have lately appeared in the *Practitioner* Mr. Alexander Foulerton has undertaken a careful and most destructive criticism of the parasitic hypothesis of cancer causation. He concludes (1) that a hypothetical parasite does not in any important way elucidate the pathology of carcinoma; (2) that so far as theoretical considerations go, the balance of probabilities is against the theory that carcinoma is caused by a parasite from without; (3) that there is no evidence that any of the parasites which have been described as the cause of carcinoma have any causal relation to the disease; (4) that while we are ignorant as to the immediate causation of cancer, the practically undisputed fact that the epithelial cells, whose vicious proliferation produces the obvious phenomena of carcinoma, are capable of migration from their normal environment and of self multiplication when in abnormal surroundings, renders any parasitic theory unnecessary.

What then has he to offer in place of the dethroned parasitic hypothesis? In answer to this he propounds what he speaks of as the "vital theory of the causation of carcinoma." The two outstanding facts bearing upon the causation of carcinoma are: first, the fact, practically undisputed at the present time, that the essential cells of secondary foci of new growth are directly derived from the cells of the primary tumour, and these latter in their turn from normal epithelium; and secondly, that carcinoma is a disease which, with extremely rare exceptions, occurs only in adults. The basis of the vital theory of carcinoma is that the essential cell of a carcinoma is a normal epithelial cell removed from its normal environment, still capable of active growth and reproduction, but parasitic in its habit of life for the time being, inasmuch as it can serve no useful purpose in the corporate economy and maintains its existence at the expense of the working tissues. If such a normal epithelial cell once penetrates below its basement membrane and gains access to the lymph channels of the sub-epithelial connective tissue there is no possible reason why it should not be swept along with the lymph stream, no possible reason why it should not reproduce itself in any foreign tissue in which it may happen to come to rest. Under what conditions, then, may an epithelial cell penetrate its basement membrane and gain access to the lymph channels? and what is it that in children inhibits the further proliferation of such intruding epithelial cells? With regard to the first question there is but little to be said; we must be content to know that under some conditions such penetration does occur. That being so the remaining problem is: Why is it that the epithelial cell, having left its normal environment, is unable to maintain an independent existence in the foreign tissues of the child as it can in those of the adult?

In considering the relative activities of the several groups of tissues at different ages we may roughly divide the post-natal term of life into three periods. First there is a period of activity on the part of the mesoblastic tissues, resulting in the growth of the framework of the body; then there is a period in which the activity of the epithelial cells and mainly of those of the epiblastic layer is the main feature; and, finally, there is the stage of degeneration. The predominating activities of different ages evidently affect in a special degree different sorts of tissue, and one has to inquire what is the cause of the gradual access of activity which occurs to the epithelial cells of hitherto resting hair follicles, to the epithelium of the testicles, and to that of the ovary and mammary gland in the female after the coming of puberty? On the answer to this question may depend the answer to the other question: What is the cause of that epithelial activity which enables epithelial cells to penetrate their basement membrane and form tumours in the deeper tissues—a process which also only occurs after puberty? Clearly, says Mr. Foulerton, there are two possible reasons. Either with puberty, fresh stimuli, specific for the growth of epithelial cells, come into existence, or old influences inhibitory of the activity of epithelium are ceasing to operate. Again, just as there must be some cause for the access of activity to epithelial tissues at this period of life, so there must be some cause for the slacking off in the activity of growth of the mesoblastic tissues; and this also may be due either to a cessation of a stimulating or to a development of some inhibitory influence. Thus it would appear that the cause of cancer is to be sought among those influences which lead the activities of different kinds of tissue to predominate at different times of life, those vital causes which enable epithelial tissue, either by dint of its own exuberance of growth or from the lowered resistance of the mesoblastic tissues, to intrude among the latter and give rise to carcinomatous tumours and secondary growths. Now as a basis of experimental research it has been provisionally assumed that the immunity of children from carcinoma and the reason of the inactivity during childhood of such epithelial tissue as is not immediately concerned in the nutrition of the body, may depend upon the presence in the serum of some "anti-epithelial" body or bodies, and that these may be formed as the result of a tissue reaction provoked by the presence in the serum of such substances as, for example, the metabolic products of the thymus gland, an organ which is largely developed in infancy but gradually diminishes in size and importance as life goes on; for if the immunity of the child against proliferation of epithelial cells in tissues to which such cells are naturally foreign were due to such tissue reaction, then the peculiar age incidence of carcinoma would be readily explicable by facts which are known in connection with antibacterial immunity. In connection with this theory the apparent activity of certain of the ductless glands during early life and their atrophy with the oncoming of puberty seemed to be of importance. Hence during the past two years experiments with thymus gland products have been carried out. That the treatment founded upon this hypothesis has not, so far, resulted in any permanent good, in the advanced cases in which it has been tried, by no means detracts from the ingenuity or the probability of the theory.

